# Terameprocol, a methylated derivative of nordihydroguaiaretic acid, inhibits production of prostaglandins and several key inflammatory cytokines and chemokines

**DOI:** 10.1186/1476-9255-6-2

**Published:** 2009-01-08

**Authors:** D Eads, RL Hansen, AO Oyegunwa, CE Cecil, CA Culver, F Scholle, ITD Petty, SM Laster

**Affiliations:** 1 Department of Microbiology, North Carolina State University, Raleigh, NC 27695, USA

## Abstract

**Background:**

Extracts of the creosote bush, *Larrea tridentata*, have been used for centuries by natives of western American and Mexican deserts to treat a variety of infectious diseases and inflammatory disorders. The beneficial activity of this plant has been linked to the compound nordihydroguaiaretic acid (NDGA) and its various substituted derivatives. Recently, tetra-O-methyl NDGA or terameprocol (TMP) has been shown to inhibit the growth of certain tumor-derived cell lines and is now in clinical trials for the treatment of human cancer. In this report, we ask whether TMP also displays anti-inflammatory activity. TMP was tested for its ability to inhibit the LPS-induced production of inflammatory lipids and cytokines *in vitro*. We also examined the effects of TMP on production of TNF-α in C57BL6/J mice following a sublethal challenge with LPS. Finally, we examined the molecular mechanisms underlying the effects we observed.

**Methods:**

RAW 264.7 cells and resident peritoneal macrophages from C57BL6/J mice, stimulated with 1 μg/ml LPS, were used in experiments designed to measure the effects of TMP on the production of prostaglandins, cytokines and chemokines. Prostaglandin production was determined by ELISA. Cytokine and chemokine production were determined by antibody array and ELISA.

Western blots, q-RT-PCR, and enzyme assays were used to assess the effects of TMP on expression and activity of COX-2.

q-RT-PCR was used to assess the effects of TMP on levels of cytokine and chemokine mRNA.

C57BL6/J mice injected *i.p. *with LPS were used in experiments designed to measure the effects of TMP *in vivo*. Serum levels of TNF-α were determined by ELISA.

**Results:**

TMP strongly inhibited the production of prostaglandins from RAW 264.7 cells and normal peritoneal macrophages. This effect correlated with a TMP-dependent reduction in levels of COX-2 mRNA and protein, and inhibition of the enzymatic activity of COX-2.

TMP inhibited, to varying degrees, the production of several cytokines, and chemokines from RAW 264.7 macrophages and normal peritoneal macrophages. Affected molecules included TNF-α and MCP-1. Levels of cytokine mRNA were affected similarly, suggesting that TMP is acting to prevent gene expression.

TMP partially blocked the production of TNF-α and MCP-1 *in vivo *in the serum of C57BL6/J mice that were challenged *i.p*. with LPS.

**Conclusion:**

TMP inhibited the LPS-induced production of lipid mediators and several key inflammatory cytokines and chemokines, both *in vitro *and *in vivo*, raising the possibility that TMP might be useful as a treatment for a variety of inflammatory disorders.

## Background

The creosote bush, *Larrea tridentata*, is common in the Sonoran deserts of Mexico and the American southwest. The Pima, Yaqui, Maricopa and Seri tribes have used various extracts and preparations from this plant to treat a wide variety of disorders [1,2]. The leaves can be used in a bath for chicken pox or rheumatism, while a decoction made from the boiled leaves is used as a poultice for skin sores. Skin sores can also be treated with a powder made from dried leaves and stems. The leaves can be used to make a tea (chaparral tea) that is used to treat many disorders including cancer, venereal disease, tuberculosis, colds, and rheumatism. Consumption of high levels of *L. tridentata *can cause hepatic necrosis [3,4], although damage is temporary and reversed when *L. tridentata *is withdrawn from the diet.

The leaves and stems of *L. tridentata *contain high quantities of the phenolic compound nordihydroguaiaretic acid (NDGA), a lipophilic anti-oxidant that has been used as a preservative in fats and oils. Many of the medicinal effects of *L. tridentata *have also been attributed to the effects of this compound [2]. NDGA has been shown to inhibit 5-lipoxygenase activity *in vitro *[5,6], and experiments have shown that it inhibits neutrophil production of LTB_4 _[7,8], degranulation [7,8], phagocytosis [9], and the respiratory burst [9]. NDGA affects levels of intracellular calcium [10,11], as well as exerting effects on mitochondria [12,13], and the Golgi complex [14-16]. NDGA has been shown to exert anti-tumor effects [17] and to block apoptosis induced either by tumor necrosis factor-α (TNF) [18-21] or CD95 ligand [22,23].

*L. tridentata *leaves also contain 3-O-methyl NDGA, with one methyoxl and three hydroxyl side chains rather than the four hydroxyl groups found on NDGA [24]. 3-O-methyl NDGA has been shown to inhibit replication of a number of strains of HIV and prevent both basal transcription and Tat-regulated transactivation *in vitro *[24]. This effect arises from the ability of 3-O-methyl NDGA to interfere with the binding of the transcription factor Sp1 to the long terminal repeats of HIV, an effect that was not seen with NDGA itself [24]. Based on these results, eight distinct methylated forms of NDGA were tested for their effects on HIV Tat-mediated transactivation [25]. The results of this investigation revealed that tetra-O-methyl NDGA (also known as M4N or terameprocol (TMP)) displayed the highest level of anti-HIV activity [25]. TMP has also been shown to block the replication of herpes simplex virus *in vitro *[26] and to inhibit transcription from the early promoter P_97 _of human papillomavirus 16 in transfected cells [27]. Both effects were again attributed to the ability of TMP to interfere with the binding of Sp1 to DNA. TMP has been found to arrest the growth of certain tumor-derived cell lines in the G_2 _phase of the cell cycle by inhibiting production of cyclin-dependent kinase cdc2 mRNA [28]. Experiments *in vivo *with mice, with a number of different tumor-derived and transformed cell lines, revealed a similar growth inhibitory effect resulting from decreased gene expression of both cdc2 and survivin [28,29] leading to the suggestion that TMP may be useful in humans to treat cancer. Indeed, three clinical trials with TMP to treat human tumors have been completed and two more are now underway (Clinicaltrials.gov database, accessed 5/28/08).

In this report we have investigated a novel role for TMP; as an inhibitor of inflammation. We reasoned that TMP might have anti-inflammatory activity since many of the disorders for which *L. tridentata *is traditionally used contain an inflammatory component. In this manuscript we have focused on TMP's ability to inhibit production of inflammatory lipids and cytokines from macrophages and macrophage-like cells. The results of our experiments reveal inhibition of both cytokine and lipid mediator production and suggest that multiple molecular mechanisms underlie these effects. Overall, our data suggest that TMP may be useful in clinical situations to treat a variety of inflammatory disorders.

## Methods

### Cells and Media

RAW 264.7 cells were obtained from the American Type Culture Collection (Manassas, VA) and were cultured in Dulbecco's Modified Eagle's (DME) Medium with 4 mM L-glutamine, 4.5 g/L glucose, 1.5 g/L sodium bicarbonate with 10% FCS. Most media and supplements were obtained from Sigma-Aldrich, St. Louis, MO. FCS was obtained from Atlanta Biologicals, Atlanta, GA. For production of cell culture supernatants, 1.5 × 10^5 ^cells/well were plated in 24 well tissue culture plates in 1 ml culture media. Following treatment, supernatants were collected, centrifuged for 2 min at 8,000 rpm to remove debris, aliquoted and stored at -80°C. Normal resident peritoneal macrophages were obtained from 8–10 week old C57BL6/J mice (Charles River Laboratories, Inc. Wilmington, MA). Peritoneal lavage was performed with DME serum-free media. Following washing, the resulting cells were plated in DME with 10% FCS, incubated overnight, and then washed to remove non-adherent cells.

### Chemicals and Biological Reagents

Unless otherwise indicated, reagents were purchased from Sigma-Aldrich, St. Louis, MO. TMP was supplied by Erimos Pharmaceuticals, Raleigh, NC. DMSO was used as the solvent for TMP in all experiments except for those performed *in vivo *with mice. The maximum DMSO concentration was 1.0% in all assays. This concentration of DMSO was tested in all assays and did not affect the results. LPS from *Salmonella *Minnesota R595 was purchased from LIST Biological Laboratories, Inc. (Campbell, CA).

### ELISA kits

PGE_2_, 6-keto-PGF_1α_, MCP-1, IL-12/23 p40, RANTES, and TNF-α ELISA kits were purchased from R&D Systems (Minneapolis MN), Assay Designs (Ann Arbor, MI), eBioscience (San Diego, CA), or USBiological (Swampscott, MA). The PGF_2α _kit was purchased from Assay Designs and the IL-23p19 kit was purchased from eBioscience. All lipid mediator kits are competitive type immunoassays while the cytokine kits are direct capture assays. In each case, sample values were interpolated from standard curves. Optical density was determined using a PolarStar microplate reader (BMG Labtechnologies, Durham, NC).

### Cytokine arrays

For cytokine analysis, the RayBio Mouse Inflammation Antibody Array I was purchased from RayBiotech, Inc., Norcross, GA. According to manufacturer's instructions, the array membranes were incubated with blocking buffer followed by undiluted culture supernatants for 1.5 h. Then, the membranes were washed, incubated with biotin-conjugated Abs for 1.5 h and HRP-conjugated strepavidin for 2 h. The membranes were next incubated in detection buffer and exposed to X-ray film. Finally, scans of the X-ray films were analyzed with Photoshop (Adobe) to determine spot density.

### Intraperitoneal challenge with LPS

Animal experiments were carried out in accord with approved IACUC protocol. Each group of experimental animals consisted of 5, 6–8 week old, 15–16 g C57BL6/J mice (Charles River). The groups received i.p. injections of either PBS, hydroxypropyl-β-cyclodextrin with PEG 300 (CPE) vehicle [30], 20 μg of LPS in CPE vehicle, 1 mg of TMP in CPE vehicle, or 20 μg of LPS and 1 mg TMP in vehicle. CPE vehicle and TMP/CPE vehicle injections were administered 1 h prior to LPS or PBS injections. Injection volumes were 100 μl for TMP and vehicle and 200 μl for LPS and PBS. The mice were monitored for 3 hours, sacrificed, and blood collected by cardiac puncture. Serum was separated and levels of TNF-α, PGE_2_, and MCP-1 determined by ELISA.

### Collection of peritoneal macrophages

Macrophages were collected by peritoneal lavage from 6–8 week old C57BL6/J mice (Charles River).

After collection the cells were centrifuged, counted and plated at 2 × 10^5 ^per well in 24 well tissue culture plates. The cells were allowed to adhere for 2–4 hr, washed to remove non-adherent cells and then treated as described within 24 h.

### Quantitative RT-PCR assays

Total RNA of treated and untreated cells was extracted using the RNAeasy kit (Qiagen, Valencia, CA) according to manufacturer's specifications. Residual genomic DNA was eliminated by using on-column DNase digestion with the RNase-free DNase set (Qiagen) and resulting extracts were resuspended in nuclease free water. Total amount and purity of RNA was determined using a Nanodrop 1000 spectrophotometer (ThermoFisher Scientific, Waltham, MA). Total RNA (1 μg) was denatured and reverse transcription was performed with the Improm ll reverse transcription kit (Promega, Madison, WI) in a reaction mix containing random hexamers as primers (50 ng/μl) for 60 min at 42°C. The iQTM SYBR Green supermix kit (BioRad, Hercules, CA), was used for Real-time PCR analysis, cDNA was amplified using primers specific for murine GAPDH, TNF-α, MCP-1, RANTES and COX-2. Primer combinations are GAPDH [antisense: 5' ATG TCA GAT CCA CAA CGG ATA GAT 3'; sense: 5' ACT CCC TCA AGA TTG TCA GCA AT 3']; TNF-α [antisense: 5' AGA AGA GGC ACT CCC CCA AAA 3'; sense: 5' CCG AAG TTC AGT AGA CAG AAG AGC G 3']; MCP-1 [sense: 5' CAC TAT GCA GGT CTC TGT CAC G 3'; antisense: 5' GAT CTC ACT TGG TTC TGG TCC A 3']; RANTES: [sense: 5' CCC CAT ATG GCT CGG ACA CCA 3'; antisense: 5' CTA GCT CAT CTC CAA ATA GTT GAT 3']; COX-2: [sense: 5' GCA TTC TTT GCC CAG CAC TT 3'; antisense: 5' AGA CCA GGC ACC AGA CCA AAG A 3']. All primer pairs were purchased from Integrated DNA Technologies, Coralville, IA. Cycling conditions for all PCRs are available upon request.

PCR was performed in 96 well plates (Eppendorf AG, Hamburg, Germany). Samples were amplified for a total of 50 cycles, followed by a meltcurve analysis to ensure the specificity of reactions. To generate a standard curve, total RNA was isolated from the cells and 300–600 bp fragments of the gene of interest were amplified by RT-PCR using cognate primer sets. PCR fragments were gel purified, quantified and the copy number was calculated. Serial ten fold dilutions were prepared for use as templates to generate standard curves. All samples were normalized to amplified murine GAPDH. GAPDH control was analyzed per plate of experimental genes to avoid plate-to-plate variation. Final RT-PCR data is expressed as the ratio of copy numbers of experimental gene per 10^3 ^copies of GAPDH for samples performed in duplicates.

### Peroxidase Assay for the measurement of COX-2 Activity

Inhibition of the peroxidase activity of purified COX-2 enzyme was measured using a modified chromogenic assay, described previously [31], in which N,N,N',N'-tetramethyl-p-phenylenediamine (TMPD) was utilized to measure the oxidation of PGG_2 _to PGH_2_. Briefly, approximately 100 U/ml of ovine COX-2 (Cayman Chemical Co., Ann Arbor, MI) was mixed with an assay buffer containing 100 mM Tris-HCl pH 8.0, 1 μM bovine hemin and the inhibitor TMP. This mixture was incubated in a temperature controlled 1 cm glass cuvette at 25°C for 10 minutes to allow for enzyme and inhibitor equilibration. The peroxidase activity of the COX-2 enzyme was initiated by adding 100 μM arachidonic acid. TMPD (170 μM final) was added at the same time as the arachidonic acid and the reaction was monitored for six minutes using a Shimadzu UV-2401PC kinetic reading spectrophotometer (Shimadzu, Kyoto, Japan) at 611 nm. Absorbance was recorded at one second intervals using UV probe software (Shimadzu). After three minutes hydrogen peroxide was added to a final concentration of 70 μM to further catalyze the peroxidase reaction and the kinetic reading was continued for an additional three minutes. Control reactions were analyzed without inhibitor or without enzyme for comparison.

### Immunoblotting

Cell monolayers were washed twice with cold phosphate buffered saline (PBS), solubilized in lysis buffer (50 mM Hepes, pH 7.4, 1 mM EGTA, 1 mM EDTA, 0.2 mM sodium orthovanadate, 1 mM phenylmethylsulfonyl fluoride, 0.2 mM leupeptin, 0.5% SDS), and collected by scraping. The protein concentration for each sample lysate was determined using the Pierce BCA system (Pierce, Rockford, IL). Equal protein samples (15 to 30 μg) were loaded on 8% Tris-Glycine gels and subjected to electrophoresis using the Novex Mini-Cell System (Invitrogen). Following transfer, blocking and probing, bands were visualized using the SuperSignal Chemiluminescent system (Pierce, Rockford, IL). Scans of films were then analyzed with Photoshop (Adobe) to determine band density.

### [^3^H]AA-release assays

2.5 × 10^4 ^cells were plated into 24-well flat-bottom tissue culture plates (Fisher Scientific, Pittsburgh, PA) and labeled overnight with 0.1 μCi/ml [^3^H]AA. The following morning, the cells were washed 2× with Hank's balanced salt solution (HBSS), allowed to recover for an additional 2 h, and washed again prior to treatment. At indicated time points, 275 μl aliquots of media were removed from the wells and centrifuged to remove debris. 200 μl of the supernatant was removed for scintillation counting (LS 5801, Beckman, Fullerton, CA) and total [^3^H]AA-release was calculated by multiplying by a factor of 2. Each point was performed in triplicate and maximum radiolabel incorporation was determined by lysing untreated controls with 0.01% SDS and counting the total volume.

### Influenza A virus propagation

Influenza A/PR/8/34 (VR-1469) was purchased from the American Type Culture Collection (Manassas, VI) and propagated in MDCK Cells (ATCC CCL-34). T-75 flasks of cells at 90% confluency were inoculated with 0.01 MOI of virus in 2 ml of Virus Growth Medium (VGM) made up of DMEM containing 0.2% BSA, 25 mM Hepes buffer, 100 U/ml Penicillin, 100 μg/ml Streptomycin, and 2 μg/ml TPCK-treated Trypsin (LS003740, Worthington-Biochem, Lakewood, NJ). Viral supernatants were harvested at 36 to 48 h, centrifuged to remove cellular debris, and supplemented with BSA to a final concentration of 0.5%. Aliquots were frozen and stored at -80°C. Titers of influenza A virus were determined by plaque assay using MDCK cells. Briefly, 200 μl of serially diluted virus in VGM was inoculated onto confluent MDCK cells in 24-well plates. After a 30 min absorption period, 0.8 ml of overlay was added (0.6% Tragacanth in VGM). After 48 h of incubation the overlay was removed, the cells washed with cold PBS, fixed with cold acetone:methanol (1:1), and stained with crystal violet.

### Statistical analyses

Statistical analyses were performed with PRISM^® ^software (Graphpad Software, San Diego, CA). Significant differences between means were determined using unpaired Student's t-tests with 95% confidence intervals.

### Assay for cell proliferation

To evaluate the effects of TMP on cell proliferation we utilized the CyQUANT Cell Proliferation Assay Kit (Molecular Probes, Eugene OR). Briefly, cells were seeded in triplicate at a density of 5 × 10^3 ^cells/well in 96 well plates and allowed to adhere for 24 h. Treatments were then performed and the plates processed according to manufacturer's instructions. The fluorescence intensity of CyQUANT GR dye, which is proportional to cellular DNA content, was then measured using the PolarStar microplate reader (BMG Labtechnologies, Durham, NC).

### Assay for apoptosis

An assay for active caspase-3 (Cayman Chemical Co., Ann Arbor MI, #10009135) was used to monitor the apoptosis-inducing activity of TMP. Briefly, RAW 264.7 cells were plated in 96 well tissue culture plates and treated with TMP for 24 h. Then, according to the manufacturer's instructions, the medium was removed, cells washed and lysis buffer added. A substrate for active caspase 3 (N-Ac-DEVD-N-MC-R110) was then added which, when cleaved by caspase 3, generates a fluorescent product with an emission maximum of 535 nm. Positive and negative controls were supplied by the manufacturer. All points were performed in triplicate and values shown are means +/- SEM.

## Results

### TMP and prostaglandin production

**Figure 1:**
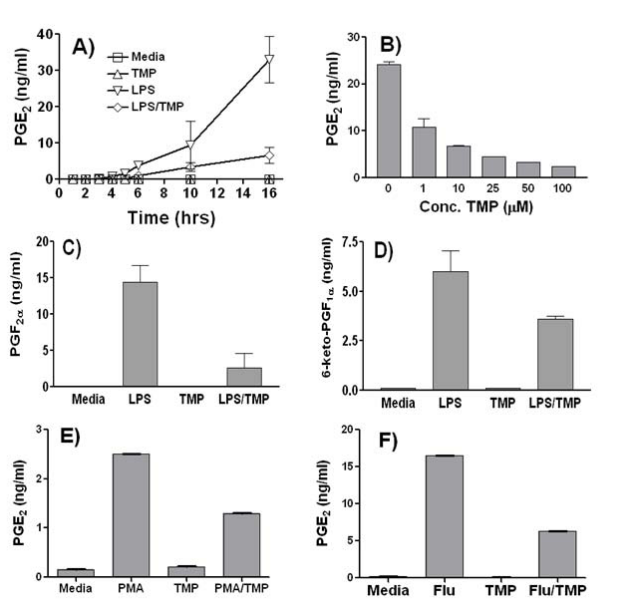
**Inhibition of prostaglandin production by TMP**. RAW 264.7 cells were incubated with LPS and/or TMP for the indicated times and then PGE_2 _concentrations in culture supernatants were determined by ELISA (A). RAW 264.7 cells were incubated with LPS in the presence of increasing concentrations of TMP and PGE_2 _concentrations were determined by ELISA (B). RAW 264.7 cells were left untreated (Media) or treated with LPS and/or TMP and the concentrations in culture supernatants of PGF_2α _(C) and 6-keto-PGF_1α _(D) were determined by ELISA. RAW 264.7 cells were left untreated (Media) or treated with PMA (10 ng/ml) (E) or influenza A virus PR/8/34 (5 pfu/cell) (F) in the presence or absence of TMP and PGE_2 _concentrations in culture supernatants were determined by ELISA. Unless otherwise indicated concentrations of LPS and TMP were 1 μg/ml and 25 μM, respectively, and the incubation time was 16 h. Panels A and B show representative experiments while the other panels show the mean ± SEM from 3 experiments. All samples were assayed in duplicate and error bars are less than symbol size where not shown. TMP was added simultaneously in experiments with LPS or PMA while TMP was added 30 m after infection with influenza A. In panels C-F, asterisks indicate significant differences between treatments with inducing agents alone and inducing agents with TMP (p < 0.05, Student's t-test).

The goal of this set of experiments was to determine whether TMP can inhibit production of prostaglandins from RAW 264.7 macrophage-like cells. These cells have been used extensively as a model for prostaglandin production by primary macrophages [32-34]. As shown in Fig. 1A, we found that treatment of RAW 264.7 cells with 1 μg/ml of LPS induced robust PGE_2 _production. PGE_2 _was first detected 4–6 h after treatment with LPS began, and levels continued to rise during the remainder of the treatment period. Fig. 1A also shows that TMP at 25 μM strongly inhibited production of PGE_2_. This effect was apparent early and maintained throughout the 16 h incubation period. As shown in Fig. 1B, we found that TMP displayed concentration-dependent inhibition of prostaglandin production. Typically, a 10 μM concentration of TMP inhibited PGE_2 _production by approximately 60% while levels of inhibition reached 80–90% with 25 μM TMP.

Our experiments showed that the inhibitory effect of TMP was not selective for production of PGE_2_. As shown in Figs. 1C and 1D, 25 μM TMP inhibited the LPS-induced production of PGF_2α _and PGI_2_/prostacylin (as measured by production of the PGI_2 _hydration product 6-keto-PGF_1α_). In addition, we found that the inhibitory effects of TMP are not specific for LPS-induced prostaglandin production. TMP inhibited production of PGE_2 _when PMA (Fig. 1E) or the influenza A virus PR/8/34 (Fig. 1F) were used as agonists. Finally, it should be noted that in all the experiments shown in Fig. 1, TMP and LPS were added simultaneously to the RAW 264.7 cells. Several experiments were performed in which the RAW 264.7 cells were pretreated with TMP (for up to several hours) but we did not find any enhanced suppression of PGE_2 _production following treatment with LPS (data not shown).

### TMP and its effects on the expression and activity of COX-2

**Figure 2:**
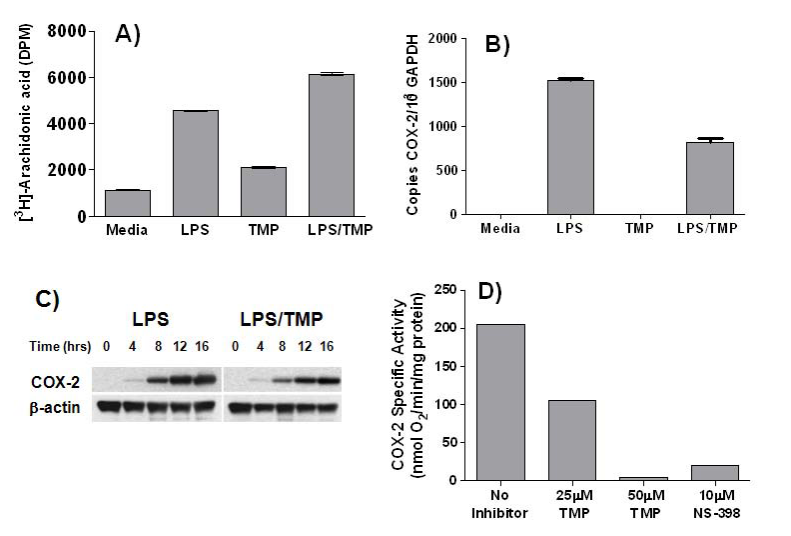
**The effects of TMP on expression and activity of COX-2**. RAW 264.7 cells were labeled overnight with [^3^H]-arachidonic acid, washed, then either left untreated (Media), or treated with LPS (1 μg/ml) and/or TMP (25 μM) for 16 h (A). Supernatants were collected and radioactivity determined by scintillation counting. RAW 264.7 cells were treated with LPS (1 μg/ml) alone or in combination with TMP (25 μM) and copy number of COX-2 mRNA determined by q-RT-PCR as described in the Materials and Methods (B). RAW 264.7 cells were treated with LPS (1 μg/ml) and/or TMP (25 μM), and the expression of COX-2 protein was examined by Western blot (C). TMP was added to purified placental ovine COX-2 protein and specific activity determined as described in the Materials and Methods (D).

Our next set of experiments was designed to understand the molecular mechanism by which TMP inhibited prostaglandin production. TMP's ability to inhibit the production of different prostaglandins, and to inhibit the production of PGE_2 _induced by different agonists, suggested that TMP was likely acting on a common, downstream element of the prostaglandin biosynthetic pathway, such as cytosolic phospholipase A_2 _(cPLA_2_) [35] or COX-2 [36,37]. As shown in Fig. 2A, we found that TMP failed to inhibit the LPS-induced release of [^3^H]-arachidonic acid from prelabeled cells. In fact, [^3^H]-arachidonic acid release was actually enhanced by TMP. These results are consistent with TMP exerting a block in arachidonic acid metabolism downstream from cPLA_2_. Therefore, a series of experiments was performed to examine the effects of TMP on the expression and activity of COX-2. Initially, we examined the effects of TMP on the expression of COX-2 mRNA. As shown in Fig. 2B, we found that TMP reduced the LPS-induced expression of COX-2 mRNA, and a deficit of approximately 40% was evident after a 16 h treatment with LPS. However, the significance of this finding is unclear. As shown in Fig. 2C, we found that TMP caused only an approximate 20% reduction in COX-2 protein expression under the same conditions. Finally, we also tested whether TMP could directly inhibit the enzymatic activity of COX-2. An assay was established in which the activity of purified ovine COX-2, alone or in the presence of inhibitors, could be measured spectrophotometrically. As shown in Fig. 2D, we found that the activity of COX-2 was inhibited by 40–50% in the presence of 25 μM TMP while inhibition of COX-2 activity was essentially complete in the presence 50 μM TMP. The level of inhibition of COX-2 activity by 50 μM TMP was comparable to that observed in the presence of 10 μM NS-398, a well characterized inhibitor of COX-2 [38].

### The effects of TMP on cytokine production

**Figure 3:**
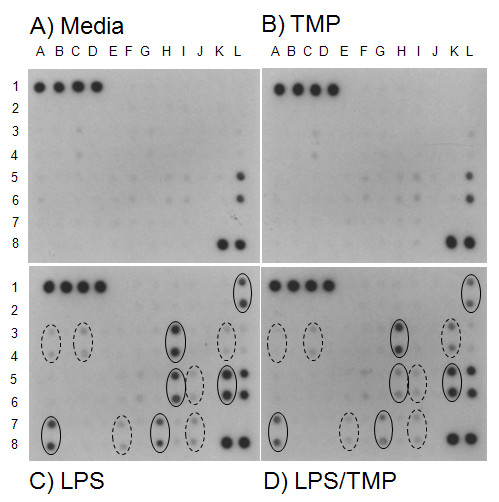
**The effects of TMP on cytokine production**. RAW 264.7 supernatants were collected and assayed for cytokine production using the Mouse Cytokine Array I (RayBiotech, Norcross GA). Shown in this figure are scans of films developed from array filters following incubation with supernatants from either untreated control cells (A), or from cells following incubation with 25 μM TMP (B), 1 μg/ml LPS (C), or 25 μM TMP and 1 μg/ml LPS (D). All supernatants were collected following a 16 h incubation period. The cytokines, chemokines, growth factors, and inflammatory products detected by the array and their respective coordinates are: Eotaxin, H1/2; Eotaxin-2, I1/2, Fas Ligand, J1/2; Fractalkine, K1/2; GCSF, L1/2; GM-CSF, A3/4; IFN-γ, B3/4; IL-1α, C3/4; Il-1β, D3/4; IL-2, E3/4; IL-3, F3/4; IL-4, G3/4; IL-6, H3/4; IL-9, I3/4; IL-10, J3/4; IL-12p40p70, K3/4; IL-12p70, L3/4; IL-13, A5/6; IL-17; B5/6; I-TAC, C5/6; KC, D5/6; Leptin, E5/6; LIX, F5/6; Lymphotactin, G5/6; MCP-1, H5/6; M-CSF, I5/6; MIG, J5/6; MIP-1α, K5/6; MIP-1γ, L5/6; RANTES, A7/8; SDF-1, B7/8; TCA-3, C7/8; TECK, D7/8; TIMP-1, E7/8; TIMP-2, F7/8; TNF-α, G7/8, sTNF R1, H7/8; sTNF R2, I7/8. Positive controls are located at positions A1, B1, C1, D1, K8, and L8. Negative controls are located at positions A2, B2, C2, and D2. Blanks are located at positions E1, E2, J7, J8, K7, and L7. Solid and dashed ellipses indicate the coordinates of cytokines and chemokines induced by LPS to high and low levels, respectively, as discussed in the text.

**Figure 4:**
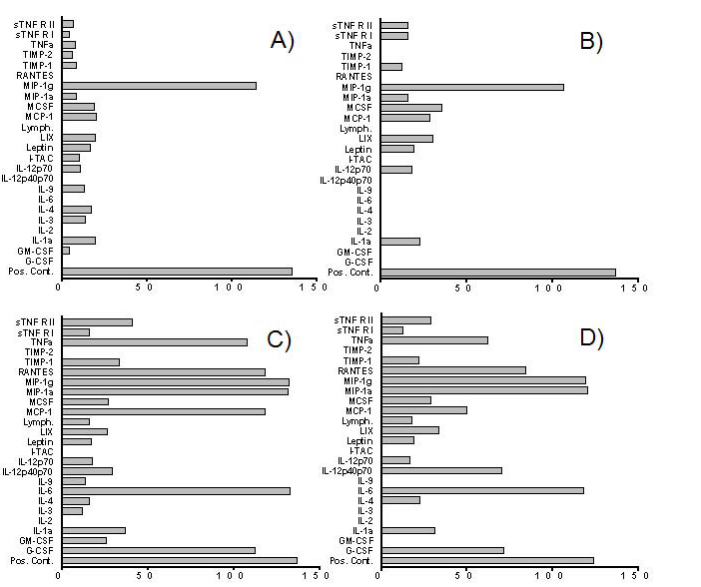
**The effects of TMP on cytokine production**. Images of the arrays shown in Fig. 3 were analyzed using Photoshop (Adobe) and mean pixel intensity (x-axis) determined for each array position. Supernatants were collected from untreated control cells (A), or from cells following incubation with 25 μM TMP (B), 1 μg/ml LPS (C), or 25 μM TMP plus 1 μg/ml LPS (D). Mean intensity values are plotted for the 24 products which were detected under one or more of the experimental conditions. SEM was less than 5% for each pair of array positions.

Macrophage derived cytokines are critical to a variety of inflammatory processes and, therefore, we sought to evaluate TMP's effect on cytokine production from RAW 264.7 cells. First, an antibody filter array was used to survey the effects of TMP on cytokine production. The array we used (mouse inflammatory antibody array 1, RayBiotech, Norcross, GA) simultaneously detects 21 cytokines and/or growth factors and 15 chemokines. The array also contains antibodies for tissue inhibitor of metalloprotease-1 (TIMP-1) and -2 (TIMP-2) and for soluble TNF receptors 1 (sTNF R1) and 2 (sTNF R2). Images of representative arrays are shown in Fig. 3, while semi-quantitative data derived from these arrays are shown in Fig. 4. As shown in Figs. 3A and 4A, only the cytokine MIP-1γ (coordinates L5 and L6) was detected at substantial levels in supernatants from unstimulated RAW 264.7 cells. We also found that treatment with TMP itself did not exert a strong effect on this profile (Figs. 3B and 4B). Subtle changes, both increases and decreases, were seen in the levels of several cytokines and again only MIP-1γ was detected at high levels.

As expected, we found that stimulation of RAW 264.7 cells with 1 μg/ml LPS dramatically enhanced the production of a number of cytokines and chemokines (Figs. 3C and 4C). For purposes of discussion we have divided these into two groups. One group of cytokines and chemokines was induced to high levels, with mean pixel densities within 75% of the positive controls included with the array kit. The coordinates on the array of these cytokines and chemokines are enclosed by solid ellipses in Fig. 3. Included in this group are RANTES (A7/8), TNF-α (G7/8), IL-6 (H3/4), MCP-1 (H5/6), MIP-1α (K5/6), and G-CSF (L1/2). A second set of cytokines, including; GM-CSF (A3/4), IL-1α (C3/4), M-CSF (I5/6), and IL-12p40p70 (K3/4) was induced to a lesser degree. The coordinates of these cytokines are enclosed by dashed ellipses in Fig. 3. Mean pixel densities for cytokines in this group were typically between 20–25% above negative controls. Finally, we found that LPS also triggered an increase in the production of TIMP-1 (E7/8) and sTNF R2 (I7/8).

As shown in Figs. 3D and 4D, we found that TMP exerted a range of effects on LPS-induced cytokine production. Among the cytokines normally induced by LPS to high levels; TMP produced two levels of suppression. Very slight suppression was noted for two cytokines, IL-6 (11%) and MIP-1α (8%), while substantially higher levels of suppression were noted for RANTES (29%), G-CSF (36%), TNF-α (43%), and MCP-1 (58%). TMP also exerted a range of effects on the cytokines produced at lower levels. Production of GM-CSF was blocked completely, while slight suppression was noted for IL-1α (16%). In contrast, secretion of M-CSF was increased to a small degree (7%), and that of IL-12p40p70 was increased substantially (141%). TMP also inhibited production of TIMP-1 (36%) and sTNF R2 (30%).

**Figure 5:**
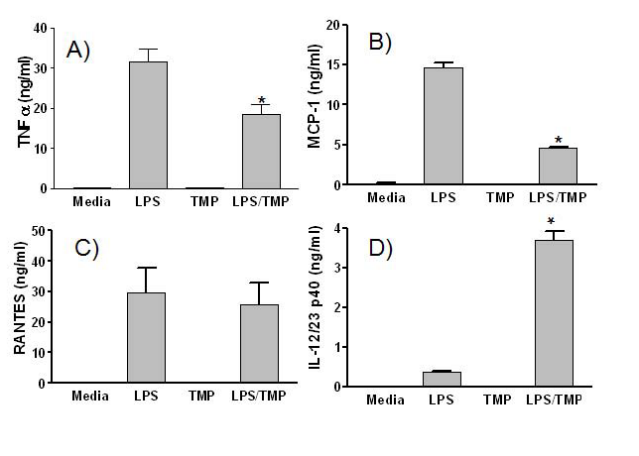
**The effects of TMP on cytokine production measured by ELISA**. RAW 264.7 cells were left untreated (Media), or treated with either 1 μg/ml LPS and/or 25 μM TMP for 16 h. Culture supernatants were then analyzed by ELISA for TNF-α (A), MCP-1 (B), RANTES (C), or IL-12/23 p40 (D). All samples were assayed in duplicate and values shown are means ± SEM from 2–3 independent experiments. Asterisks indicate significant differences between treatments with LPS alone vs. LPS with TMP (p < 0.05, Student's t-test).

Since antibody filter arrays are typically semi-quantitative, we sought to confirm several of the effects we had noted using specific cytokine ELISAs. As shown in Fig. 5A, suppression of TNF-α production measured by ELISA (42%) very closely matched the level of suppression observed on the array (43%). The suppressive effects of TMP were also very similar for MCP-1 production, when measured by ELISA (Fig. 5B) (67%) or by the array (58%). On the other hand, ELISA did not confirm the inhibition of RANTES production (Fig. 5C) noted on the array. At present, the reason for this discrepancy is unclear. Finally, we also used ELISA to investigate the TMP-dependent increase in IL-12p40p70. The increase in IL12p40p70 noted on the array, in the absence of an increase in IL-12p70 (Figs. 3D and 4D), suggests that TMP enhances the LPS-dependent production of p40 monomers or homodimers. Alternatively, it is also possible that this represents production of IL-23 since p40 is also a component of IL-23. As shown in Fig. 5D, an ELISA specific for IL-12p40 confirmed the finding from the array. However, an ELISA specific for IL-23 (n = 3, 10 pg/ml sensitivity) did not detect any of this cytokine (data not shown). We conclude, therefore, that these supernatants contain either monomers or homodimers of p40.

### TMP and its effects on cytokine mRNAs

**Figure 6:**
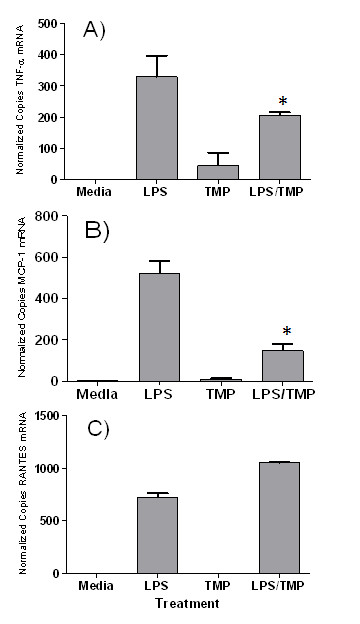
**The effects of TMP on copy number of cytokine mRNA**. RAW 264.7 cells were left untreated (Media), or treated with LPS (1 μg/ml) and/or TMP (25 μM). After 16 h, total RNA was extracted from the cell pellet and mRNA copy number determined by q-RT-PCR for TNF-α (A), MCP-1(B) and RANTES (C). Panels show means ± SEM of representative experiments with duplicate copy number determinations performed in each experiment. Asterisks indicate significant differences between treatments with LPS and LPS/TMP (p < 0.05, Student's t-test).

Next, a series of experiments was performed to define the mechanism by which cytokine production was inhibited by TMP. Specifically, we used quantitative RT-PCR to investigate the effects of TMP on production of cytokine mRNA. As shown in Fig. 6, we found a strong correlation between the effects of TMP on cytokine protein levels, as measured by ELISA, and expression of cytokine mRNA. Levels of TNF-α protein and mRNA were reduced by 42 and 40%, respectively; while levels of MCP-1 protein and mRNA were reduced by 67 and 76%, respectively. Similarly, neither RANTES mRNA (Fig. 6C) nor protein (Fig. 5C) levels were suppressed by TMP. In fact, we measured a small increase in RANTES mRNA following treatment with TMP and LPS (Fig. 6C).

### The effect of TMP on production of PGE_2_, cytokines, and chemokines by peritoneal macrophages

**Figure 7:**
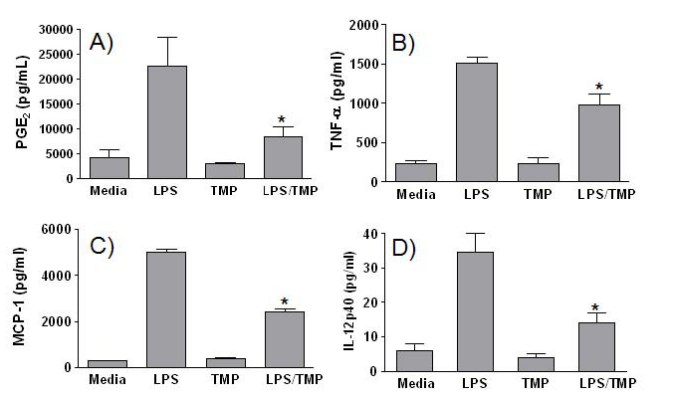
**The effects of TMP on cytokine, chemokine and inflammatory lipid production by peritoneal macrophages**. Macrophages were collected from C57BL6/J mice and left either untreated (Media), or treated overnight with 1 μg/ml LPS and/or 25 μM TMP. Levels of PGE_2 _(A), TNF-α (B), MCP-1 (C) and IL-12/23 p40 (D) in cell culture supernatants were subsequently determined by ELISA. Panels show means ± SEM from 2–3 experiments for each mediator. Asterisks indicate significant differences between treatments with LPS and LPS/TMP (p < 0.05, Student's t-test).

To further substantiate the results of our experiments with RAW 264.7 cells, a set of experiments was performed with normal mouse macrophages. Resident peritoneal macrophages were harvested from C57BL6/J mice, then treated with LPS and/or TMP *in vitro*, and cell supernatants were examined for PGE_2 _and several cytokines. As shown in Fig. 7, the results of these experiments were highly similar to those seen with RAW 264.7 cells. Levels of PGE_2_, TNF-α, and MCP-1 produced by peritoneal macrophages were all reduced by TMP to extents comparable to those seen in experiments with the RAW 264.7 cell line. The exception was the effect of TMP on the production of IL-12/23 p40. TMP did not enhance IL-12/23 p40 production from LPS-treated peritoneal macrophages as it did with LPS-treated RAW 264.7 cells. Instead, levels of IL-12p40 were reduced by approximately 60%.

### The effect of TMP on production of cytokines *in vivo*

**Figure 8:**
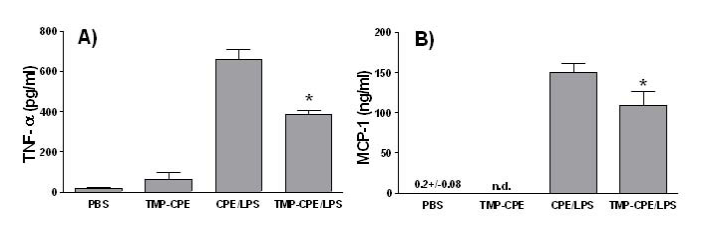
**The effects of TMP on serum levels of TNF-α and MCP-1**. C57BL6/J mice (5/treatment) were used in a transient endotoxemia model to test TMP's ability to inhibit cytokine and chemokine production *in vivo*. One group of mice was injected only with PBS while a second group received only TMP (1 mg) in the CPE vehicle. Serum was collected from these mice after 4 h. A third group received the CPE vehicle followed 1 h later by 20 μg of LPS while a fourth group received 1 mg of TMP in the CPE vehicle followed 1 hr later by 20 μg of LPS. Serum was collected from these mice 3 h after the LPS injection. Levels of TNF-α and MCP-1 were determined by ELISA. Panels show means ± SEM from representative experiments. Asterisks indicate significant differences between treatments with CPE vehicle followed by LPS and TMP in CPE vehicle followed by LPS (p < 0.05, Student's t-test). n.d. – not determined.

The finding that TMP inhibits production of TNF-α *in vitro *raises the possibility that TMP may be useful *in vivo *in a variety of inflammatory conditions. To test whether TMP can inhibit the production of TNF-α *in vivo *we established a transient endotoxemia model in C57BL6/J mice [39]. The mice were injected *i.p. *with 20 μg of LPS in the CPE vehicle which caused the animals mild distress; the mice huddled for 2–3 hours then returned to normal behavior. We also found, as has been reported [39] that this dose of LPS induced a transient increase in levels of serum TNF-α. Serum levels of TNF-α peaked 2–3 h after injection with LPS and returned to pre-injection levels by 1–2 h later (data not shown). Two experiments were then performed in which TMP was administered in the CPE vehicle followed 1 h later by LPS. Serum was collected 3 h after the LPS challenge and levels of TNF-α were determined by ELISA. The results from the first of these experiments are shown in Fig. 8A. As expected, we measured low levels of TNF-α in the serum of mice that received PBS (19 ± 3 pg/ml; mean ± SEM), CPE vehicle (60 ± 9 pg/ml), or TMP in the CPE vehicle (49 ± 9). Much higher levels of TNF-α were measured in mice first treated with the CPE vehicle followed by LPS in PBS (657 ± 50); and, strikingly, we found that TMP offset this increase by 41% (385 ± 19 pg/ml).

We also examined these serum samples for PGE_2 _using an ELISA kit that permits measurements of PGE_2 _in mouse serum (#P9053-30, USBiological, Swampscott MA). The results of these assays did not reveal any significant changes in PGE_2 _concentration in any treatment group. Levels of PGE_2 _varied from 2.5–5.0 ng/ml per mouse in the PBS injected mice and remained in that range in groups treated with TMP in CPE vehicle, CPE vehicle followed by LPS, and TMP in CPE vehicle followed by LPS (data not shown).

A second experiment was then performed to confirm the effects of TMP on production of TNF-α. Overall, the results were highly similar to those in the first experiment. We found low levels of TNF-α in the serum of mice treated with PBS (21 ± 21 pg/ml), higher levels with LPS treatment following installation of the CPE vehicle (430 ± 39) and significant suppression with TMP (42%) (251 ± 52 pg/ml, p < 0.05, Student's t-test). In this experiment, rather than test for PGE_2_, we quantified levels of MCP-1. Our results showed significant suppression (27%) by TMP of the LPS-induced accumulation of MCP-1 in serum (Fig. 8B).

### The effects of TMP on the growth of RAW 264.7 cells

**Figure 9:**
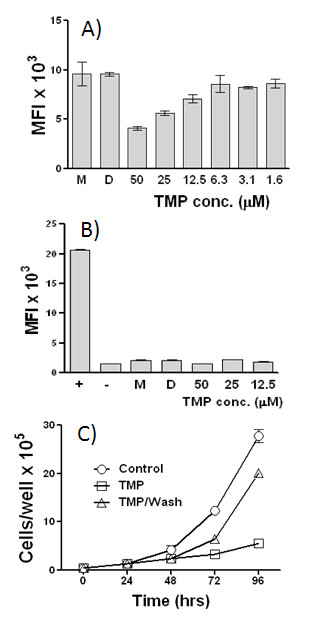
**The effects of TMP on the growth and viability of RAW 264.7 cells**. TMP was added to RAW 264.7 cells *in vitro *and their growth monitored for 24 h using the CyQuant proliferation assay (A). The induction of apoptosis in RAW 264.7 cells following a 24 h exposure to TMP was measured using an assay for active caspase 3 (Cayman) (B). In panels A and B, M and D indicate media and DMSO controls. In panel B, + and – indicate the addition of positive and negative controls supplied by the manufacturer. In C, cells were plated in 24 well plates, allowed to adhere 24 h, then washed and incubated with either fresh medium or medium containing 25 μM TMP. After 24 h, the medium containing TMP was removed from one set of wells, replaced with fresh medium without TMP, and the cells were incubated for an additional 48 h. Cell counts were determined by hemocytometer. Values shown are means +/- SEM and all points were performed in triplicate.

Experiments summarizing the effects of TMP on the growth of RAW 264.7 macrophage-like cells are shown in Fig. 9. Using an assay that monitors DNA accumulation (Cyquant) (Fig. 9A) we found that the growth of RAW 264.7 cells was inhibited at the higher concentrations of TMP tested. For example, growth of RAW 264.7 cells was inhibited by approximately 40% during a 24 h incubation with 25 μM TMP. In contrast, as shown in Fig. 9B, we did not detect any apoptosis at this concentration of TMP. The lack of toxicity of TMP towards RAW 264.7 cells was confirmed in experiments where RAW 264.7 cells were transiently exposed to 25 μM TMP. As shown in Fig. 9C, when TMP is withdrawn following a 24 h exposure, the cells quickly regain their normal rate of growth.

## Discussion

Chronic activation or hyper-activation of the innate immune system is the cause of many damaging inflammatory and auto-immune pathological reactions. The secretory products of activated macrophages are major contributors to these reactions. Our goal in these studies was to test whether TMP can inhibit the production of macrophage derived pro-inflammatory cytokines and lipids. Our impetus was twofold; to better understand the mechanisms underlying the traditional anti-inflammatory uses for *L. tridentata*, and to determine whether a safe, potentially effective anti-cancer drug might have an alternative use. The results of our experiments showed that TMP can indeed inhibit the production of several key macrophage products. Production of prostaglandins was suppressed as was the production of certain cytokines and chemokines raising the possibility that it may indeed be useful to treat inflammation. Clearly, further study will be necessary to determine the extent to which naturally occurring tetra-O-methyl NDGA contributes to the anti-inflammatory activity found in extracts of *L. tridentata*.

Excess prostaglandin production has been linked to a variety of inflammatory responses and auto-immune pathological reactions. PGE_2_, for example, has been linked to production of amyloid-β peptides in Alzheimer's disease [40], while both PGE_2 _and PGI_2_/prostacyclin have been linked to joint destruction during rheumatoid arthritis [41,42]. Our experiments revealed broad suppression of prostaglandin production by TMP *in vitro *with levels of PGE_2_, PGF_2α_, and PGI_2_/prostacyclin all being reduced. These results are consistent with those of early studies which showed that NDGA, the parent compound of TMP, could suppress prostaglandin production by primary murine macrophages [43,44]. Our results suggested that TMP may indeed be useful clinically to treat a number of disorders and, therefore, we investigated the mechanism underlying this activity. Because prostaglandin production was suppressed regardless of the inducing agent (LPS, phorbol ester, or influenza virus), we reasoned that it was unlikely that TMP was interfering with signals from specific receptors such as TLR-4 [35]. Therefore we focused our investigation on common, downstream elements in the prostaglandin pathway such as cPLA_2 _[35], and COX-2 [36,37]. TMP did not inhibit the LPS-induced release of [^3^H]-arachidonic acid, which suggested that it was not affecting the activity of cPLA_2_, and therefore we focused our efforts on COX-2. Our experiments revealed a number of effects of TMP on COX-2, including; a 40% reduction in COX-2 mRNA, a 20% reduction in COX-2 protein, and inhibition of COX-2 enzymatic activity. The effects of TMP on the expression of COX-2 mRNA were not entirely surprising since the 5' flanking region of the COX-2 gene has 3 Sp1 binding sites [45] and TMP is known to inhibit Sp1 binding to DNA [24]. Exactly how TMP's effect on the expression of COX-2 mRNA ultimately impacts prostaglandin production is not clear. The decrease in COX-2 mRNA resulted in only a modest decrease in COX-2 protein making it unlikely that this effect of TMP is a major determinant of its ability to suppress prostaglandin production. Rather, it seems that a direct inhibitory effect of TMP on COX-2 enzymatic activity is primarily responsible for the suppression of prostaglandin synthesis. This result was unexpected. Although NDGA, the parent compound of TMP, is widely known as an inhibitor of lipoxygenases, direct inhibition of COX-2 activity by NDGA appears not to have been described. In addition, lipoxygenase inhibition by NDGA is thought to depend on its anti-oxidant activity, but this is greatly reduced in TMP, which carries methyl rather than hydroxyl groups. Clearly, more studies need to be performed to examine the interaction between COX-2 and TMP and elucidate the mechanism of inhibition.

Our results suggest that TMP is a potent, direct inhibitor of COX-2 activity, and the magnitude of this effect may be sufficient to fully account for the suppression of prostaglandin production by TMP in LPS-stimulated cells. However, since the yield of the various prostaglandins tested was not affected equally by a fixed concentration of TMP, it is conceivable that TMP may also affect the activity of one or more of the prostaglandin synthases downstream from COX-2. Further research will be required to evaluate this possibility.

In this study we also addressed the effects of TMP on the LPS-induced production of cytokines and chemokines by RAW 264.7 cells. The antibody array we used revealed increases in the production of 10 cytokines or chemokines following treatment with LPS. We also measured increased levels of TIMP-1 and sTNF R2 in culture supernatants. Among these molecules, TMP failed to suppress production of the chemokine MIP-1α, or the cytokines IL-6, IL-1α, and M-CSF. On the other hand, suppression was noted for the chemokine MCP-1, and for the cytokines TNF-α, G-CSF, and GM-CSF. We also noted suppression of production of TIMP-1 and sTNF R2. In contrast to the results obtained for COX-2, we found a strong correlation between the levels of cytokine mRNAs and their respective protein products. TMP suppressed the accumulation of TNF-α and MCP-1 mRNA and protein to similar extents, while it had no effect on the accumulation of RANTES mRNA or protein. The suppression of cytokine mRNA accumulation by TMP in LPS-stimulated cells may be determined by its ability to inhibit binding of the Sp1 transcription factor to DNA. Experiments with monocytic cell lines have shown that Sp1 binding sites are required for the LPS-induced activation of the TNF-α promoter [46], whereas they are completely dispensable for activation of the RANTES promoter [47]. Although a direct effect of TMP on cytokine gene transcription could account for the reduced accumulation of mRNAs, other, post-transcriptional mechanisms cannot be excluded. For example, TNF-α production can be regulated at the level of mRNA stability [48], as well as by protease cleavage of the TNF-α precursor [49], and either of these might be affected by TMP. Interestingly, if TMP inhibited the expression or activity of the TNF-α converting enzyme, it would be expected to reduce the levels of both secreted TNF-α [49] and sTNF R2 [50], as we observed in our experiments. Additional investigation will be required to fully elucidate the mechanisms by which TMP suppresses the production of various cytokines including its potential effects on other transcription factors linked to inflammation, such as NF-κB.

Overall, for testing the effects of TMP on cytokine production, the RAW 264.7 cell line proved to be an excellent model, although we did find one major difference in its response to LPS compared to that of peritoneal macrophages. Treatment with TMP caused an increase in the expression of IL-12/23 p40 in LPS-stimulated RAW 264.7 cells, but this effect was not seen with LPS-stimulated peritoneal macrophages. In peritoneal macrophages, we observed a response that was consistent with the effects of TMP on other cytokines; namely inhibition of expression. Why the LPS-dependent expression of IL-12/23 p40 is increased by TMP in RAW 264.7 cells is not clear. Apparently, the pathway(s) that regulate the expression of IL-12p40 are altered in RAW 264.7 cells so that TMP causes an enhancing effect.

Our experiments also revealed that TMP inhibited the growth of RAW 264.7 cells. Based on the ability of TMP to inhibit the growth of certain tumor-derived cell lines, and its effects on Sp1, its effect on the growth of RAW 264.7 cells was not surprising. We did not detect any apoptosis and the growth inhibitory effect disappeared when the TMP was withdrawn, indicating that TMP is not damaging towards RAW 264.7 cells and is likely causing reversible, cell cycle arrest. In the future it will be interesting to determine whether there is a link between the growth inhibitory effects of TMP and the ability of TMP to inhibit production of cytokines and inflammatory lipids in RAW 264.7 cells. However, since peritoneal macrophages do not grow *in vitro*, we can conclude that growth inhibition is not a prerequisite for TMP to exert its anti-inflammatory effects.

Based on the ability of TMP to inhibit cytokine and lipid mediator production *in vitro*, we tested whether TMP could exert these effects *in vivo*. We used a mouse model of endotoxemia in which a sublethal dose of LPS is administered *i.p. *resulting in transient increases in the serum levels of several cytokines. Increased cytokine levels are evident within one hour; continue to rise for 2–3 hrs, then return to pretreatment levels 1–2 hrs later. The response to LPS under these circumstances is complex and cytokine production involves many cell types from different organs and tissues. LPS is transported from the peritoneum to the liver via the portal vein [51] where both hepatocytes and Kupffer cells respond by producing TNF and other cytokines [52]. Likewise, spleen, brain and bone marrow cells also produce cytokines following *i.p. *exposure to LPS [52-54]. Whether these cells are responding directly to the LPS or to the cascade of cytokines produced by the liver is not entirely clear. Pharmacokinetic experiments in mice indicate that TMP also spreads rapidly through the body following *i.p. *administration. Park, et al. [29] have shown that 4 hours after a single *i.p. *injection of TMP (2 mg) it can be readily detected in a variety of tissues and organs (liver, adipose tissue, brain, etc.) at μM concentrations comparable to those we utilized *in vitro*. TMP-mediated suppression of cytokine production *in vivo *may therefore be occurring at a variety of anatomical locations outside the peritoneal cavity and defining these sites will be the focus of future investigations.

It has been over two decades since TNF-α was identified as one of the major mediators of endotoxemia and cachexia [55]. Since then, TNF has been linked to a number of auto-immune and inflammatory disorders including; rheumatoid arthritis, inflammatory bowel disease, and psoriasis, to name a few (reviewed in [56]). Although expensive and difficult to administer, TNF-α blockers such as Infliximab, Etanercept, or Adalimumab have proven to be clinically effective for the management of these disorders [57]. The results of our experiments showed that TMP can reduce levels of TNF-α *in vivo*. While it is clear that many additional experiments remain to be performed, our results raise the possibility that TMP may be useful for treating inflammatory diseases that are mediated by TNF-α. Furthermore, the use of TMP may be advantageous over that of other TNF-α blockers because TMP also inhibits the production of other cytokines and inflammatory lipids. Elevated levels of MCP-1, for example, have been linked to psoriasis [58], while production of PGE_2 _contributes to rheumatoid arthritis [59]. In both of these diseases TMP might prove more effective than drugs that target only TNF-α.

In summary, we have examined the ability of TMP to inhibit the secretion of cytokines, chemokines, and inflammatory lipids from activated macrophages. Our results show that TMP can inhibit production of both prostaglandins and several key inflammatory cytokines and chemokines. Therefore, TMP could potentially be used as a treatment for a number of different inflammatory disorders.

## Conclusion

• TMP inhibited production of prostaglandins from LPS-stimulated RAW 264.7 cells and from murine peritoneal macrophages.

• The ability of TMP to inhibit prostaglandin production was linked to effects on levels of COX-2 mRNA and protein and to inhibition of COX-2 enzymatic activity.

• TMP inhibited production of several key inflammatory cytokines and chemokines by RAW 264.7 cells and murine peritoneal macrophages.

• The ability of TMP to inhibit cytokine and chemokine production was correlated with effects on levels of cytokine and chemokine mRNA.

• TMP reduced levels of TNF-α and MCP-1 in the serum of mice challenged *i.p. *with a sublethal dosage of LPS.

• The ability of TMP to inhibit production of both protein and lipid mediators of inflammation suggests that it may have broad clinical application for the treatment of inflammatory and autoimmune disorders.

## Abbreviations

TMP: terameprocol; LPS: lipopolysaccharide; TNF-α: tumor necrosis factor-α; COX-2: cyclo-oxygenase-2; ELISA; enzyme-linked immunosorbent assay; q-rt-PCR: quantitative reverse transcriptase polymerase chain reaction; MCP-1: monocyte chemotactic protein-1; NDGA: nordihydroguaiaretic acid; HIV: human immunodeficiency virus; CPE: 20% hydroxypropyl beta-cyclodextrin and 50% polyethylene glycol 300; PMA: phorbol-12-myristate 13-acetate.

## Competing interests

Erimos Pharmaceuticals produces TMP and would stand to benefit financially if TMP was used clinically to treat inflammation. None of the authors is paid by Erimos nor do they have stock or shares in the company. S.M.L. has a patent pending for the use TMP to treat inflammation associated with influenza infection but this application does not include claims relating to bacteria or bacterial products.

## Authors' contributions

D.E., R.L.H. and C.A.C were responsible for investigating the effects of TMP on prostaglandin, cytokine and chemokine production. These individuals were responsible for all ELISA's and cytokine arrays.

C.E.C was responsible for defining the effects of TMP on COX-2 enzyme activity.

A.O.O. was responsible for investigating the effects of TMP on the expression of COX-2, cytokine, and chemokine mRNA.

F.S., I.T.D.P., and S.M.L. participated in design and coordination of the study, acquisition of funding, and drafting of the manuscript.
